# Responses to crizotinib in patients with *ALK*-positive lung adenocarcinoma who tested immunohistochemistry (IHC)-positive and fluorescence *in situ* hybridization (FISH)-negative

**DOI:** 10.18632/oncotarget.10560

**Published:** 2016-07-13

**Authors:** Di Ma, Zheng Wang, Lin Yang, Xinlin Mu, Yan Wang, Xinming Zhao, Junling Li, Dongmei Lin

**Affiliations:** ^1^ Department of Medical Oncology, Cancer Institute & Hospital, Chinese Academy of Medical Sciences, Beijing, China; ^2^ Department of Pathology, Beijing Hospital of the Ministry of Health, Beijing, China; ^3^ Department of Pathology, Cancer Institute & Hospital, Chinese Academy of Medical Sciences, Beijing, China; ^4^ Department of Respiratory and Critical Care Medicine, Peking University People's Hospital, Beijing, China; ^5^ Department of Diagnostic Radiology, Cancer Institute & Hospital, Chinese Academy of Medical Sciences, Beijing, China; ^6^ Key Laboratory of Carcinogenesis and Translational Research (Ministry of Education), Department of Pathology, Peking University Cancer Hospital & Institute, Beijing, China

**Keywords:** non-small-cell lung cancer, ALK status, crizotinib, immunohistochemistry, fluorescence in situ hybridization

## Abstract

Although the Ventana immunohistochemistry (IHC) platform for detecting anaplastic lymphoma kinase gene (*ALK*) (D5F3) expression was recently approved by the US Food and Drugs Administration (FDA), fluorescence *in situ* hybridization (FISH) is still the “gold-standard” method recommended by the US National Comprehensive Cancer Network (NCCN) guideline for NSCLC. We evaluated 6 *ALK*-positive lung adenocarcinoma patients who tested Ventana IHC-positive and FISH-negative and assessed their clinical responses to the *ALK* tyrosine kinase inhibitor (TKI) crizotinib. Histologic and cytologic specimens from the 6 patients were stained with Ventana anti-*ALK*(D5F3) rabbit monoclonal primary antibody using the OptiView™ DAB IHC detection kit and OptiView™ amplification kit on a Ventana BenchMark XT processor. In addition, they were also tested by FISH, qRT-PCR, next-generation sequencing (NGS), and RNAscope ISH analysis. All patients received crizotinib treatment and their follow-up clinical data were recorded. The objective response rate achieved with crizotinib therapy was 66.7% (4/6 partial responses and 2/6 stable disease). One patient in whom a new fusion type (*EML4*->EXOC6B->*ALK* fusion) was identified obtained a partial response. These findings indicate that patients with *ALK*-positive lung adenocarcinoma who test Ventana IHC-positive and FISH-negative may still respond to crizotinib therapy.

## INTRODUCTION

The anaplastic lymphoma kinase (*ALK*) tyrosine kinase inhibitor (TKI) crizotinib was approved by the US Food and Drug Administration (FDA) in 2011 for the treatment of patients with advanced non-small-cell lung cancer (NSCLC) who harbor *ALK* gene rearrangements. Consequently, the *ALK* gene fusion test is very meaningful for NSCLC patients in clinical practice. The fluorescence *in situ* hybridization (FISH) assay is recommended for the detection of *ALK* rearrangements in the US National Comprehensive Cancer Network (NCCN) guideline for NSCLC [1], and this test is still considered the “gold standard”’, whereas immunohistochemistry (IHC) was considered a screening test for this purpose [2, 3]. However, staining of specimens with the Ventana IHC ALK(D5F3) system and analyzed with OptiView™ (Roche) has been found to detect *ALK* rearrangements with more sensitivity and specificity compared with FISH or other IHC assays [4–6], and this platform was approved by the Chinese Food and Drug Administration (CFDA) in 2013. The interpretation of Ventana IHC ALK(D5F3) staining was found to display excellent inter-reader agreement in recent studies [7, 8].

Despite the high concordance of IHC, FISH, and other test methods for demonstrating the *ALK* status, almost all studies have found discrepancies in the *ALK* status with the different methods [3, 9]. Clinicians may therefore be confused when encountering discrepancies in the *ALK* status between different test platforms. To further address this issue, we collected data on 6 patients with lung adenocarcinomas who were Ventana IHC ALK(D5F3)-positive and FISH-negative from 3 hospitals in Beijing, and assessed these patients clinically following the administration of crizotinib therapy.

## RESULTS

### Clinicopathologic characteristics

The clinicopathological characteristics of the 6 patients evaluated in the study are shown in Table 1. The patients’ median age was 54 years (range, 31–69 years), and the median Eastern Cooperative Oncology Group performance score (ECOG PS) was 0 (range, 0 to 2). All patients had stage IV disease at the time crizotinib treatment was initiated, and all tumors were adenocarcinomas with no *EGFR*/*KRAS* mutations. Two patients received crizotinib as first-line treatment, while 4 had been heavily pretreated prior to the initiation of crizotinib. The median duration of crizotinib therapy was 9.71 months, and 5 patients were still receiving crizotinib at the last follow-up.

**Table 1:** Patient characteristics (n = 6)

Patient No./Gender	Age (y)	Smoking History	No. of Prior Regimens	PFS (months)	Assessment	Follow-up
1. Female	31	Never smoked	5	7.46+	Partial response	Alive
2. Male	48	Ever smoker	0	11.96+	Stable disease	Alive
3. Female	49	Never smoked	10	19.94+	Stable disease	Alive
4. Male	59	Ever smoker	2	6.60+	Partial response	Alive
5. Male	69	Ever smoker	6	15.08+	Partial response	Alive
6. Female	65	Never smoked	0	3.58	Partial response	Dead

PFS, progression-free survival; +, no progressive disease at the final assessment.

At the last follow-up date, no progressive disease was observed in 5 patients (Patients 1-5) who were all still receiving treatment at this time. However, 1 patient (Patient 6) died suddenly (due to pulmonary embolism or sudden cardiac death) after >3 months of treatment.

### Responses to crizotinib treatment

**Figure 1:**
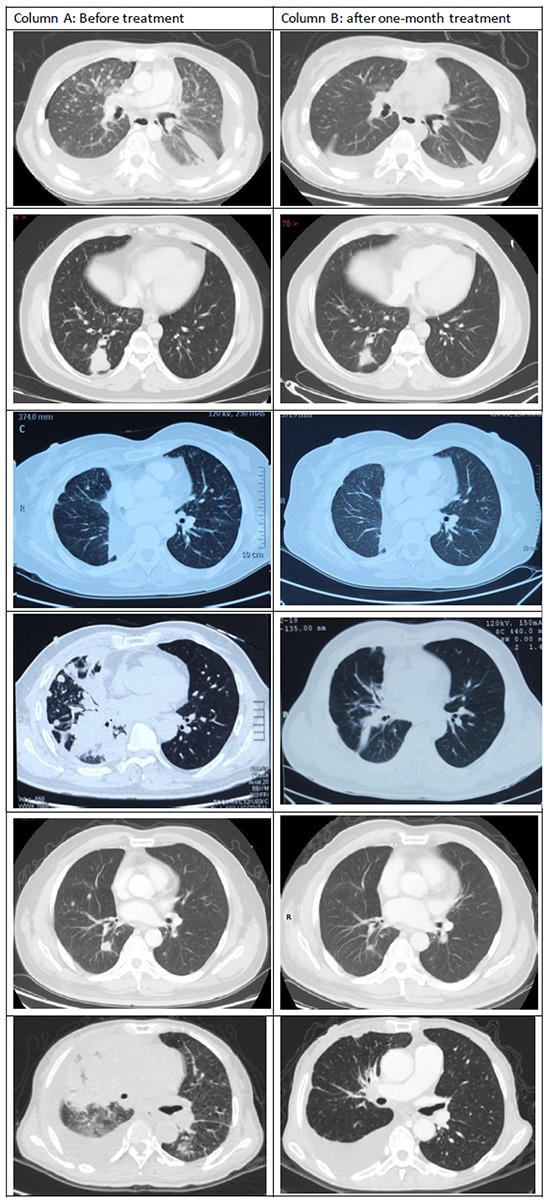
Comparisons of initial computed tomography (CT) images of the thorax with CT images at 1 month after initiation of crizotinib treatment Compared with the initial CT images (Column A), repeated CT imaging at 1 month after the initiation of crizotinib treatment (Column B) showed dramatic shrinking of the cancers in Patients 1, 3, 4, and 5.

Tumor responses to crizotinib treatment are shown in Figure 1. Four patients had a partial response and 2 had stable disease. Thus, the objective response rate achieved with crizotinib was 66.7%. In 1 patient in whom a novel fusion variant (*EML4*->*EXOC6B*->*ALK* fusion) was identified (see further below), a partial response was recorded.

No grade 4 or 5 adverse events were observed with crizotinib therapy. Although QT prolongation in 1 patient led to a temporary withdrawal of treatment and a dosage reduction, none of the patients required permanent discontinuation of the drug.

### Diagnosis of histologic and cytologic samples by IHC staining

All 6 histologic and cytologic samples were identified as CK7, TTF-1, and napsin A expression-positive by IHC staining. One of the cytologic samples showed co-expression of P63 and TTF-1, but the other 5 patients were P63-negative. Antibodies for differentiating malignant mesothelioma (CK5, WT-1, D2-40, calretinin, and desmin) were negative in tumor cells from 1 of the pleural effusion samples, but P53 expression was detected in 4 of the 6 patients. The proportions of tumor cells in the tested samples are shown in Table 2.

**Table 2:** Pathologic characteristics and molecular test results in the 6 patients

No.	Sample Types	TTF1 IHC	P63 IHC	EGFR/KRAS Mutations	ALK IHC	ALK RT-PCR	ALK FISH	RNAscope ISH	NGS-ALK	NGS ALK %	Tumor Content
P1	LB	+	-	WT/WT	+	Fusion	6%	Score 1 Score 2	E13:EXOC6B:A20	0.42% (3/710)	90%
P2	FNA	+	-	WT/WT	+	Fusion	10%	/	/	/	90%
P3	LP	+	-	WT/WT	+	/	6%	/	/	/	60%
P4	LP	+	-	WT/WT	+	/	6%	/	/	/	70%
P5	LB	+	-	WT/WT	+	/	10%	/	/	/	70%
P6	PE	+	+	WT/WT	+	Fusion	12%	Score 0 Score 2	E13:A20	15.15% (75/495)	95%

ALK FISH, % of split signals by FISH; EGFR, epidermal growth factor receptor; FISH, fluorescence *in situ* hybridization; FNA, fine needle aspiration; IHC, immunohistochemistry; ISH, *in situ* hybridization; LB, lymph nodes biopsy; LP, lung puncture; NGS, next-generation sequencing; NGS ALK (%), proportion of *ALK*-rearranged tumor cells by NGS; PE, pleural effusion; RT-PCR, reverse transcription polymerase chain reaction; Tumor content, proportion of tumor cells in tested samples; WT, wild type.

### *ALK* rearrangements by FISH

**Figure 2:**
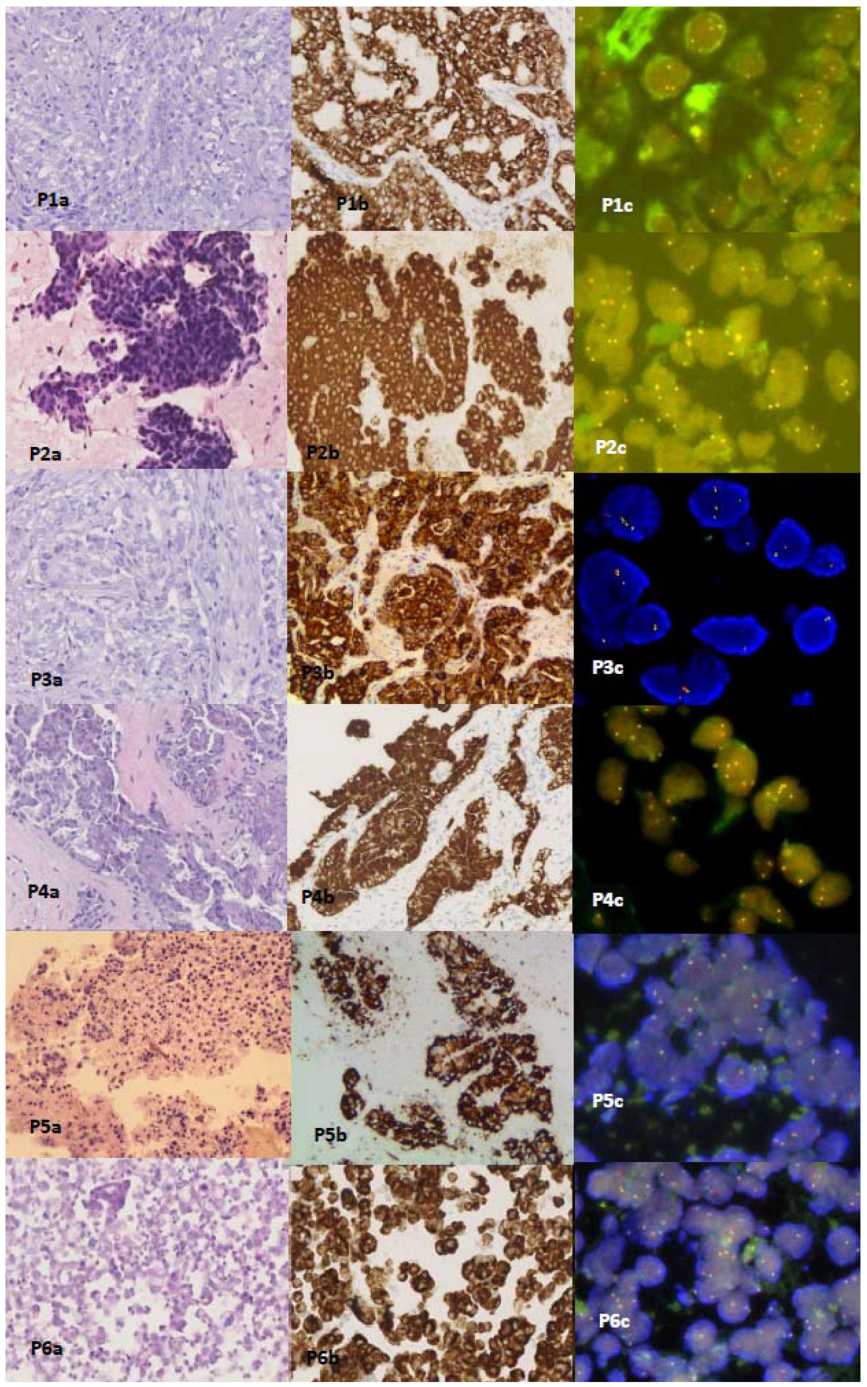
Hematoxylin and eosin (H&E) staining, Ventana IHC(D5F3) staining, and FISH staining slides from Patients 1-6 P1a-6a are H&E stained slides from histologic and cytologic samples. P2a is a fine needle aspiration (FNA) cell smear slide from Patient 2 (H&E staining × 200). P1b-6b show positive ALK protein expression by Ventana IHC ALK(D5F3) staining of sections of FFPE histologic and cytologic blocks (Ventana IHC staining × 200). P1c-6c show dual color Break Apart FISH assays of sections of the FFPE histologic and cytologic blocks from the 6 patients. Split signals (indicated by red and green signals) were under the 15% cut-off value in the 6 patients (FISH ×1000).

All cytologic and histologic samples from the 6 patients were successfully tested by FISH. The percentages of *ALK*-positive nuclei in all 6 were under 15% (range, 6% to 12%) [Table 2; Figure 2]. The *ALK* rearrangement interpretations of the FISH test results by the 3 pathologists showed concordance.

### ALK protein expression by Ventana IHC(D5F3)

Ventana IHC(D5F3) analyses of ALK protein expression were successfully performed in formalin-fixed, paraffin-embedded (FFPE) histologic and cytologic samples from all 6 patients. All of the positive signals were found to be strong and granular in the cytoplasm of adenocarcinoma tissue and cells, and they were homogeneous in staining intensity and extent. No heterogeneous tumor *ALK* expression was observed. Ventana IHC ALK(D5F3) staining results were straightforward to interpret in all 6 cases, and interpretations of the *ALK*-positive results by the 3 pathologists again showed concordance.

### *EML4-ALK* fusions by qRT-PCR

*EML4-ALK* fusions were further verified by real-time reverse transcription polymerase chain reaction (qRT-PCR) technology in 3 samples [1 histologic (Patient 1) and 2 cytologic (Patients 2 and 6)]. All 3 samples showed positive reactions in reaction tube No. 1 of the qRT-PCR kit (Table 3).

**Table 3:** *EML4-ALK* fusion types detected with the qRT-PCR kit

Tube No.	EML4-ALK Fusion Types			
1	E6;A19	E6;A20	E6ins33;A20	E6;ins18A20
	E13;A20	E13;ins69A20	E20;A20	E20;ins18A20
2	E14 ins11;del49A20	E14;del14A20	E14;del38A20	E15del60;del71A20
3	E2;A20	E2;ins117A20	E3;ins53A20	E17;ins30A20
	E17ins61;ins34A20	E17ins65;A20	E17;ins68A20	E17del58;ins39A20
	E18;A20			

### RNAscope ISH *ALK* gene RNA analysis

**Figure 3:**
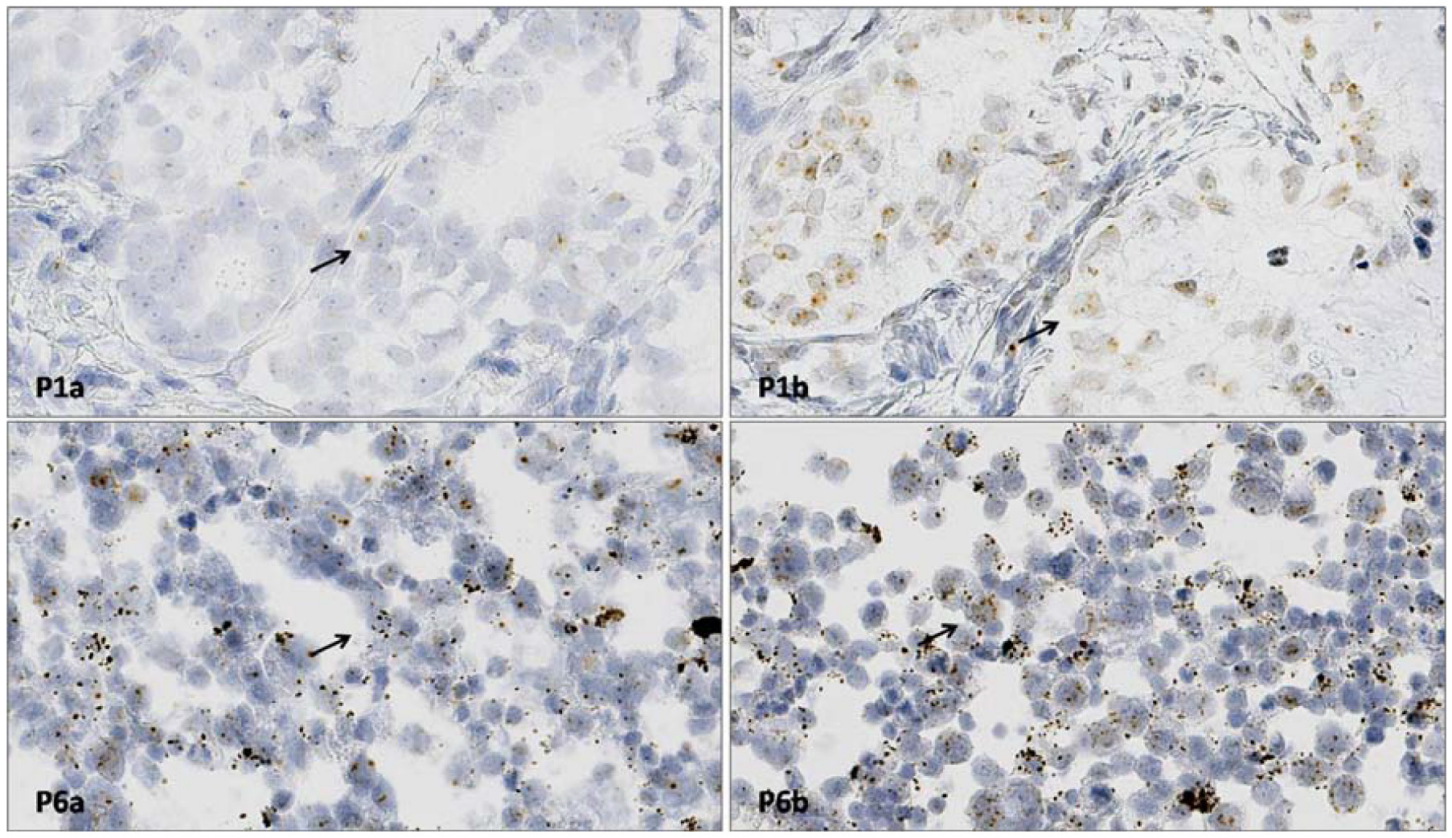
RNAscope ISH for *ALK* gene RNA detection P1a shows Hs-ALK-E1-E18 probe staining of a section from Patient 1 (score: 0). P1b shows Hs-ALK-E29-20p probe staining of a section from Patient 1 (score: 2). The black arrow shows positive signals (RNAscope ISH × 400). P6a shows Hs-ALK-E1-E18 probe staining of a section from Patient 6 (score: 1). P6b shows Hs-ALK-E29-20p probe staining of a section from Patient 6 (score: 2). The black arrow shows positive signals (RNAscope ISH × 400).

Interpretation of the RNAscope *in situ* hybridization (ISH) results for *ALK* gene RNA rearrangements indicated a score of 1 for Hs-ALK-E1-E18 and a score of 2 for Hs-ALK-19-E29-20p in Patient 1, and a score of 0 for Hs-ALK-E1-E18 and a score of 2 for Hs-ALK-19-E29-20p in Patient 6 (Table 2; Figure 3).

### Next-generation sequencing (NGS)

**Figure 4:**
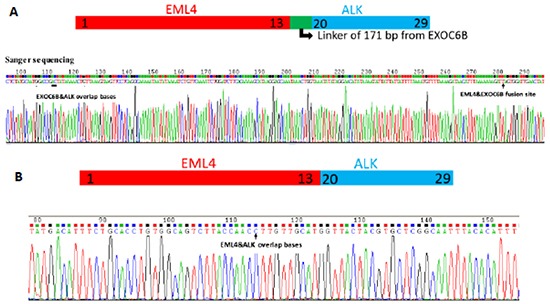
The fusion types and locations on the genome were firstly detected by cSMART technology and confirmed by Sanger sequencing with a special PCR primer pair between the flanks of the fusion site **A.** A complex fusion type, *EML4*->*EXOC68*->*AL K* was found with 171 bp insertion from *EXOC68* gene fragment in Patient 1. **B.** A familiar *EML4*->*ALK* form was found in Patient 6.

Three unique DNA templates with *EML4*->*EXOC6B*->*ALK* fusion were identified in 710 DNA copies from the tumor tissue of Patient 1. The fusion ratio was only 0.42% (3/710). After alignment with human genome (hg19), the 3 fusion DNA copies consisted of 3 gene fragments, *EML4*, *EXOC6B*, and *ALK*. Some unknown mechanisms had caused the fusion of the 3 gene fragments, and the complex fusion type and low fusion ratio caused the negative FISH results. In Patient 6, we detected 75 unique DNA templates in a total 495 DNA copies in cytologic specimens, with a fusion ratio of 15.15% (75/495). The fusion types of Patient 1 and 6 were confirmed by Sanger sequencing (Figure 4).

## DISCUSSION

Versions 3 and 4 of the NCCN guideline for NSCLC recommend IHC as the screening method for testing patients’ *ALK* status, with confirmation of positive IHC results by FISH [1]. FISH is a powerful method for detecting *ALK* gene translocations, but it is possible for the *ALK* translocation status to be missed because of variations of partners for *ALK* gene rearrangement and fusion patterns, or a low proportion of *ALK*-rearranged tumor cells in the tested tumor samples (i.e., the so-called ‘FISH borderline-positive cases’) [10]. As successive reports have revealed dramatic responses to crizotinib in *ALK*-positive NSCLC patients who tested IHC-positive and FISH-negative, the “gold-standard” place of FISH for detecting the *ALK* status has been challenged. However, insufficient clinical follow-up data have been reported in relevant studies. Only a few case reports of patients with *ALK* FISH-negative/IHC-positive results who received *ALK* inhibitor therapy were identified in a literature search, and the IHC antibodies and platforms employed were not Ventana IHC ALK(D5F3) [4, 11–13].

In this study, we evaluated 6 lung adenocarcinoma patients with FISH-negative and Ventana IHC-positive *ALK* results, 3 of whom were validated with RT-PCR technology, including 2 who were also validated with NGS and RNAscope ISH methods. As the advantage of the RNAscope method is that it can sensitively detect target RNAs in FFPE tissues, we selected two *ALK* probes that target exons 1-18 and exons 19-29, respectively, to detect the expression levels of *ALK* 5′ and 3′ regions. The RNAscope ISH results indicated that both patient samples exhibited higher RNA signals with the probe Hs-ALK-E19-E29-20p compared with Hs-ALK-E1-E18, suggesting that both patients experienced a higher expression level of the 3′ regions of *ALK*, which may have been due to *ALK* fusion causing up-regulation of RNA expression. The NGS method was performed in 2 patients (Patients 1 and 6), the results of which showed a low frequency of *ALK* gene fusion, 0.42% and 15.15%, respectively (the tumor cells in these 2 cases accounted more than 90% of the total cells examined), and revealed a novel fusion variant E13:EXOC6B:A20 in Patient 1 which was confirmed by Sanger sequencing. The cut-off value is ≥15% break-apart signals per section for interpreting FISH-positive cases, and in some studies, the percentage of *ALK* rearrangement-positive cells detected by the FISH assay has been variable, ranging from 15% to 90% [14]. It is therefore suggested that a low *ALK* gene fusion frequency may be one of the reasons for misinterpretations with the FISH method. On the other hand, the Ventana IHC, qRT-PCR, NGS, and RNAscope ISH methods are relatively sensitive, and all succeeded in detecting an *ALK* gene-positive signal.

In China, the “Standards for Diagnosis and Treatment of Primary Lung Cancer (2015 version)” guideline and the “Consensus on Diagnosis of *ALK*-positive non-small-cell Lung Cancer (2013 version)” guideline propose that FISH, Ventana IHC, and PCR-based amplification techniques are all appropriate diagnostic techniques for detecting *ALK*-positive lung cancer [15, 16]. All 6 cases of lung adenocarcinoma in our study were treated with crizotinib. Two received crizotinib as first-line treatment, while 4 had received various cytotoxic drugs (e.g., pemetrexed) and other targeted therapies previously. The longest overall survival (OS) recorded was 19.94 months at the last follow-up. The objective response rate was 66.7%, and the disease control rate was 100%. The patient in whom a novel fusion variant (E13:EXOC6B:A20) was revealed by the NGS method responded well to crizotinib treatment and obtained a partial response. Thus, our findings confirm the recommendations of the Chinese guidelines for the diagnosis of *ALK*-positive NSCLC, and the Ventana IHC ALK(D5F3) platform is now employed as a routine diagnostic tool in many hospitals in China.

The heterogeneity and abundance of NSCLC driver gene mutations has been a major concern for some time [17]. In comparison with the lower-sensitivity Sanger sequencing, more NSCLC patients with *EGFR* mutations are detected by using the relatively high-sensitivity amplification refractory mutation system (ARMS) technique. Sanger-negative and ARMS-positive patients (low abundance of *EGFR* mutations) may also benefit from EGFR-TKI therapy [18, 19]. However, as with *ALK* gene rearrangements, there have been no large-scale studies. Thus, the question as to whether there are differences in the response to ALK-TKI therapy between high-fusion frequency and low-fusion frequency populations remains unanswered. Our study demonstrated that 2 low-fusion frequency cases (NGS *ALK* gene fusion analysis) benefited from crizotinib therapy, suggesting that NSCLC patients with a low frequency of *ALK* fusion genes may also benefit from ALK-TKI treatment.

Another interesting issue is the discordant distribution of signals among cancer cells between the FISH method and the Ventana IHC ALK(D5F3) method. Why did the Ventana IHC ALK(D5F3) method show diffuse and strong granular cytoplasmic expression in a patient with a low frequency of *ALK* gene rearrangements? This phenomenon could be explained by an intercellular communication mechanism for related signal molecules [20–23]. Cell communication is a mechanism of protein molecule exchange in multicellular organisms. There are desmosomes, tight junctions, and gap junctions, which are the cellular structures or channels for intercellular communication, and there are numerous signaling molecules involved. For example, studies have shown that exosome, a molecule involved in intercellular communication, may execute the functions of intercellular protein transport, exchanges of proteins and lipids, or triggers of downstream signaling events [24]. Recently, it was found that exosome released by cancer cells is capable of transferring tumor-derived EML4-ALK rearrangement RNA to platelets [25]. It is possible that cells without *ALK* gene rearrangement are ‘infected’ with ALK fusion protein via intercellular communication, and this small amount of ‘neighbor’ protein produces a positive result with powerful signal amplification of Ventana IHC(D5F3) staining. The discrepancy of DNA and protein abnormalities remains to be explored.

In summary, the 6 FISH-negative and Ventana IHC-positive *ALK* lung adenocarcinoma cases who were treated with crizotinib responded well to the drug, with similar objective response and disease control rates to those previously reported. Our findings suggest that the FISH method cannot wholly be considered the “gold standard” for screening for *ALK* gene rearrangements in patients with NSCLC. We propose that FISH, Ventana IHC, and PCR-based amplification techniques are all appropriate as diagnostic methods for *ALK*-positive NSCLC, and a positive result with any of these methods can be used as the basis for selecting targeted therapy.

## MATERIALS AND METHODS

### Patients

Data on the 6 patients with ALK-positive metastatic lung cancer who had tested IHC-positive and FISH-negative and received treatment with crizotinib were obtained from 3 hospitals in Beijing: the Cancer Institute & Hospital, the Chinese Academy of Medical Sciences, and the Peking University People's Hospital and Beijing Hospital. The data collected included the patients’ demographic and clinicopathologic tumor characteristics, their medical and surgical treatments (including chemotherapy regimens and courses), and their responses to chemotherapy on cross-sectional imaging as assessed by the Response Evaluation Criteria in Solid Tumors (RECIST) and the National Cancer Institute Common Toxicity Criteria (for adverse events). The patients underwent clinical visits that included thoracic and abdominal computed tomography (CT) scans at baseline and after 6 to 8 weeks of crizotinib therapy. Responses were defined as the best response from the start of treatment until disease progression according to RECIST (version 1.1). Tumor assessment was reviewed by the radiologist and attending oncologist. Clinical and biologic data were collected by the treating physicians and pathologists.

The experimental use of human specimens in the study was approved by the Independent Ethics Committee of the Cancer Hospital, Chinese Academy of Medical Sciences, Beijing.

### Treatments

All patients were treated with crizotinib, which was given in a dosage of 250 mg twice daily. The occurrence of grade 4 and 5 adverse events during treatment was documented.

### Preparation of formalin-fixed, paraffin-embedded (FFPE) cytological blocks

One fine needle aspiration (FNA) sample was collected from cervical lymph nodes and fixed in 95% ethanol, and 1 pleural effusion sample with heparin added for anticoagulation was also collected. The cytologic samples were centrifuged, fixed in 4% neutral formalin (fixation time 6 hours), and embedded in paraffin. The procedures for making FFPE cytological blocks were same as for histological samples.

### IHC staining for histologic and cytologic pathological diagnosis

For pathological diagnosis, IHC antibodies were used for immunostaining of histologic and cytologic FFPE blocks. Antibodies CK7, TTF-1, napsin A, and P63 were used as markers for differentiation of lung adenocarcinoma from squamous cell carcinoma. In 1 pleural effusion sample, antibodies CK5, WT-1, D2-40, calretinin, desmin and P53 were also used as markers to differentiate lung cancer from malignant mesothelioma.

### Detection of ALK protein expression by IHC

All histologic and cytologic specimens were stained by IHC with an anti-ALK monoclonal antibody (D5F3, Roche) and tested for ALK protein expression with the OptiView^®^ DAB IHC Detection kit and the OptiView^®^ Amplification kit (Ventana Medical Systems, Inc., Tucson, AZ, USA). In accordance with the manufacturer's instruction manual, FFPE sections 4 μm thick were prepared for IHC staining, which was performed automatically using the Ventana BenchMark XT Stainer (Ventana Medical Systems Inc., Tucson, AZ, USA). The IHC stains were evaluated for expression of ALK by 3 pathologists (D.L., L.Y, and Z.W.) who were trained to identify only strong cytoplasmic granular staining in tumor cells (which was deemed a positive result).

### Detection of *ALK* rearrangement by FISH

All histologic and cytologic samples were also tested by FISH, which was carried out using the Vysis ALK Break Apart FISH Probe kit (Abbott Molecular, IL, USA). Tissue sections 4 μm thick were prepared for FISH staining, the process and interpretation of which were according to the manufacturer's instructions. Positive cases were defined as those exhibiting split signals [the 5′-part (green fluorescence) and 3′-part (red fluorescence) signals were regarded as split when the separation distance was greater than 2 fluorescence signal diameters], or an isolated red signal in more than 15% of tumor cells. At least 50 tumor cells for each section were analyzed. The interpretations of FISH stains for *ALK* rearrangement were made by 3 pathologists (D.L., L.Y, and Z.W.).

### Detection of EML4-ALK fusion by qRT-PCR

Three samples [1 histologic (Patient 1) and 2 cytologic (Patients 2 and 6)] were adequate to test for EML4-ALK fusion by qRT-PCR. In brief, total RNA was extracted from 3 slides of 4 μm thickness taken from the FFPE blocks using the AmoyDX RNA Isolation kit (Amoy Diagnostics, Xiamen, China), and mRNA was transcribed to cDNA at 42°C for 1 hour. EML4-ALK fusion was readily detected by qRT-PCR using the AmoyDx EML4-ALK Fusion Gene Detection kit (Amoy Diagnostics, Xiamen, China), according to the manufacturer's protocol.

### RNAscope ISH analysis for *ALK* gene RNA detection

Two samples [1 histologic (Patient 1) and 1 cytologic (Patient 6)] were adequate to perform RNAscope ISH analysis. FFPE tissue and cell block sections 5 μm thick were deparaffinized in xylene and then dehydrated in an ethanol series. Hybridization was with target probes (probe symbols: Hs-Probe-Hs-ALK-E1-E18 and Hs-ALK-E19-E29-20P; probe name: anaplastic lymphoma receptor tyrosine kinase). The preamplifier, amplifier, label probe, and chromogenic detection procedures were according to the manufacturer's instructions (RNAscope^®^ 2.0 HD Reagent Kit, Advanced Cell Diagnostics, Hayward, CA, USA). The scoring guidelines and interpretation were in accordance with those reported by Wang et al. [26].

### Next-generation sequencing (NGS)

Two samples [1 histologic (Patient 1) and 1 cytologic (Patient 6)] were adequate to perform NGS analysis. We used a modified circulating single molecule amplification and resequencing technology (cSMART) method, which was first described for detecting Wilson's disease in pregnant women [27]. We prepared libraries from 50 ng genomic DNA fragments with about 200 bp by ligation of universal sequencing adaptors containing unique 6 bp barcodes. Modified molecules were denatured and single strands circularized by Taq ligase. Bidirectional back-to-back primers were annealed to the 26 tiling loci in intron 19, and inverse PCR was performed to replicate targeted alleles. Amplified products were subjected to massive parallel sequencing on the MiSeq platform (Illumina Inc., CA, USA) to generate 3M paired-end reads of 2 × 200 bp. This strategy was designed to capture all possible allelic fragment sizes to minimize bias, and it provided adequate matching sequence overlap to assemble the targeted region.

To derive plasma mutation ratios, we counted only uniquely barcoded template DNA of different sizes and map positions from the preamplification library. In cases in which the start and stop sequencing bases were identical for 1 or more sequencing reads, we counted only those with a unique barcode. The final mutation ratio was determined from a minimum of 500 uniquely barcoded templates.

### Sanger sequencing confirmation of the NGS results

The precise fusion location in the human genome and partner genes was determined by cSMART technology. The special primer pair in different partner genes based on the genomic location information was designed to amplify the fusion DNA segments with polymerase chain reaction (PCR) technology, and the PCR product was analyzed by Sanger sequencing. The primer pairs were specific to DNA segments with a somatic mutation, and normal DNA segments were not enriched by PCR.

### ARMS for EGFR and KRAS mutation analysis

In all histologic and cytologic samples, EGFR gene exons 18-21 and KRAS gene exon 2 mutations were detected using the amplification refractory mutation system (ARMS). The ADx EGFR Mutations Detection kit and the ADx KRAS Mutations Detection kit (Amoy Diagnostics, Xiamen, China) were employed to perform this analytical procedure. Quantitative real-time PCR experiments were carried out using the ABI 7500 Fast PCR system (Applied Biosystems Inc., CA, USA). Ct values used to determine whether a sample was positive or negative were based on extensive validation.

### Statistical analysis

Statistical analysis was performed at the last study follow-up date (February 28, 2015) using SPSS^®^ version 17.0 (SPSS, Chicago, IL, USA). A p-value of less than 0.05 was considered to indicate statistical significance. Categorical variables were summarized with percentages, and continuous variables with medians. The patients’ progression-free survival (PFS) was calculated as the time from the date of initiation of crizotinib therapy to the first observation of disease progression, while overall survival (OS) was calculated as the time from the date of initiation of crizotinib therapy until death from any cause or until the last follow-up date.
